# Emerging Role of MicroRNA in Pancreatic Cancer

**Published:** 2012-04-26

**Authors:** Jun Xia, Fazlul H Sarkar, Zhiwei Wang

**Affiliations:** 1 Department of Biochemistry, Bengbu Medical College, Anhui, PR China; 2 Department of Pathology and Oncology, Karmanos Cancer Institute, Wayne State University, USA; 3 Department of Pathology, Beth Israel Deaconess Medical Center, Harvard Medical School, USA

**Keywords:** Pancreatic cancer, microRNA, Stem cell, Natural agents, Cancer therapy

Pancreatic cancer (PC) is of the most highly aggressive malignant disease, and currently the fourth most frequent cause of cancer-related mortality in the United States [1]. It is expected that approximately 43,920 people are diagnosed with PC and about 37,390 will die of this disease in 2012 [1]. Despite recent therapeutic advancement, the median survival rate is less than 6 months, and 5-year survival rate is less than 5%. The high mortality and poor prognosis are due to extensive metastasis, lack of early symptoms, and lack of reliable early diagnostic markers [1]. Hence, it is important to understand the molecular mechanism(s) underlying this devastating disease in order to develop new therapeutic strategies for improving the therapeutic outcome of patients diagnosed with PC.

Emerging evidence suggests that microRNAs (miRNAs) play a pivotal role in the development and progression of PC [2]. It is well documented that miRNAs are a family of the 17–25-nucleotide-long non-coding RNAs that govern the expression of approximately 30% of the protein-coding genes in human genome [2]. The miRNAs repress translation or degrade the target mRNA through binding to the 3’UTR (3’ untranslated region) of target mRNA. It is worth mentioning that miRNAs could function as either oncogenes or tumor suppressor genes, suggesting that the expression of miRNAs could be dependent on tumor types [2]. Very interestingly, one individual miRNA could regulate more than hundred mRNA targets, whereas one gene could be controlled by several miRNAs [2].

There is a wealth of literature supporting that deregulation of miRNAs are involved in the development and progression of human malignancies including PC [2]. It has been reported that the expression profiles of miRNAs could provide clues to pancreatic tumorigenesis because miRNA expression profiles could differentiate among normal pancreas, chronic pancreatitis, and PC. For example, Roldo et al. [3] found that up-regulation of miR-103 and miR-107 as well as down-regulation of miR-155 could be helpful to discriminate pancreatic tumors from the normal pancreas. We also found that miR-200 was down-regulated in the compound transgenic mice with activated K-ras and Ink4a/Arf deficiency that quickly develop pancreatic tumors [4]. Moreover, we identified up-regulation of miR-21, miR-221, miR-27a, miR-27b and miR-155, and down-regulation of miR-216a, miR-216b, miR-217 and miR-146a expression in tumors derived from this mouse model [5]. In addition, over-expression of miR-21 has been found to be associated with metastasis [3], and patients with increased miR-196a-2 have poor survival [6]. Consistent with these results, we also found that 37 miRNAs were decreased and 54 miRNAs were increased in the plasma of PC patients [7]. Specifically, miR-21 was up-regulated, whereas let-7 family and miR-146a were significantly down-regulated in the plasma of PC patients. More importantly, miR-21 expression was associated with worse survival, while let-7 expression was inversely correlated with survival [7]. These results suggest that unique miRNA signatures could be useful for the screening of high-risk patients and may serve as biological markers for disease status.

Multiple studies have demonstrated that miRNAs play pivotal roles in EMT (epithelial-to-mesenchymal transition) phenotype in PC. The EMT is a fundamental process whereby epithelial cells undergo conversion into mesenchymal cells. During EMT, cells lose epithelial characteristics with down-regulation of epithelial markers such as E-cadherin and γ-catenin, and gain the expression of mesenchymal markers including Vimentin, fibronectin, Twist, ZEB1, ZEB2, Slag, and Snail. Recently, it has been reported that miR-200 can directly target mesenchymal markers ZEB1 and ZEB2 in PC cell lines [8, 9]. Consistent with this finding, we also found that miR-200 and let-7 family was down-regulated in gemcitabine resistant PC cells that have undergone EMT [10]. Very recently, one study showed that miR-126 plays an essential role in the inhibition of invasive growth of PC cells through regulation of ADAM9 expression, leading to the induction of epithelial marker E-cadherin [11]. These published data suggest that miRNAs play critical roles in the acquisition of EMT.

Accumulating evidence suggests that miRNAs are involved in PC stem cells (CSCs). Pancreatic CSCs have been identified and isolated from human PC by flow cytometry using cell surface markers including CD44, CD24, ESA, CD133, and c-Met [12]. Recently, Ji et al. [13] identified that pancreatic CSCs have lower expression of miR-34, indicating that miR-34 could be involved in pancreatic CSCs self-renewal. Moreover, miR-34 was reported to govern pancreatic CSCs self-renewal through modulation of Notch and Bcl-2. Nalls et al. [14] reported that miR-34a is a critical regulator of pancreatic cancer progression through regulation of CSC characteristics. Moreover, differentially expressed miRNAs including miR-99a, miR-100, miR-125b, miR-192, and miR-429 have been reported in pancreatic CSCs [15]. Additionally, we demonstrated that miR-200b was down-regulated in FoxM1 over-expressing or Notch-1 over-expressing PC cell lines, which have undergone EMT consistent with CSCs phenotype [16,17]. Taken together, miRNAs have been shown to play an important role in pancreatic CSCs self-renewal.

Interestingly, altered expression of specific miRNAs has been found to be involved in drug-resistant PC cells. Previous reports from our laboratory have shown that the expression of miR-200 family and let-7 family was significantly down-regulated in gemcitabine-resistant cells [10]. Moreover, we have demonstrated that re-expression of miR-200 family resulted in increased cell sensitivity to gemcitabine [10]. In support of the role of miRNAs in drug resistance, Park et al. [18] found that anti-sense inhibition of miR-21 or miR-221 sensitizes the effects of gemcitabine in pancreatic adenocarcinoma. In agreement with this concept, Giovannetti et al. [19] showed that over-expression of miR-21 significantly suppressed anti-tumor activity by gemcitabine, suggesting that targeting miR-21 could be beneficial for overcoming gemcitabine chemo-resistance. Taken together, these data suggest that the regulation of miRNA could make drug-resistant cells to become drug-sensitive, and such strategy could be helpful to overcome drug resistance in PC.

As deregulation of miRNAs such as miRNAs over-expression or down-regulation plays critical roles in PC progression, targeting the mature miRNAs or their precursors by synthetic anti-sense olignucleotides or up-regulation of miRNAs ectopically could be a novel strategy for PC therapy. Indeed, one study revealed that transfection of the synthetic Gli-1-miRNA-3548 inhibited cell division and induced late apoptosis in PC cells [20]. Inhibition of miR-21 using anti-sense olignucleotides suppressed proliferation, and increased apoptosis in PC cell lines [18]. Similarly, over-expressions of miR-34 by using miR-34 mimics or infection with lentiviral miR-34 construct decreased cell clonogenicity and invasion as well as induced apoptosis [13]. Interestingly, several drugs have been reported to regulate the expression of miRNAs. For example, we found that metformin increased the expression of several miRNAs such as let-7a, let-7b, miR-26a, miR-101, miR-200b, and miR-200c [21].

Recently, accumulated evidence revealed that chemopreventive agents commonly known as natural compounds could target miRNAs to regulate the key cellular protein expression. Due to lack of toxicity, natural compounds have attracted more attention as chemopreventive agents to target miRNAs in recent years. We have shown that isoflavoned 3,3’-diindolylmethane (DIM) increased the expression of miR-200 family in PC cells [10]. More importantly, isoflavone or DIM treatment enhanced the sensitivity of gemcitabine-resistant cells to gemcitabine, which was in part mediated through up-regulation of miR-200 in PC cells [10]. Similarly, we demonstrated that curcumin increased PC cell sensitivity to gemcitabine through increased miR-200 and decreased miR-21 [22]. Moreover, we found that CDF, a novel synthetic analog of curcumin, inhibited the sphere-forming ability of PC cells and suppressed tumor growth, which was associated with decreased miR-21 expression and increased miR-200 expression [22]. Furthermore, we demonstrated that CDF inhibited PC cell survival, migration, invasion and CSC function in part via up-regulation of multiple tumor suppressive miRNAs including let-7 family, miR-26a, miR-101, miR-146a, and miR-200b [21]. Additionally, we found that genistein inhibited cell growth was partly mediated through up-regulation of miR-200b in Notch-1 or FoxM1 over-expressing PC cells [16,17]. These results provide experimental evidence showing that natural compounds could function as miRNA regulators, suggesting that the regulation of miRNAs by nutraceuticals could be a novel strategy for achieving better treatment outcome of patients diagnosed with PC.

Collectively, these studies clearly suggest the important roles of miRNAs in cell growth, apoptosis, invasion, metastasis, EMT, stem cells self-renewal, and drug resistance. Therefore, identification of deregulated miRNAs in PC could be useful for tumor diagnosis, disease prognosis, and for assessing therapeutic outcome. Targeting miRNAs, specifically by natural compounds could open newer avenues for the prevention of tumor progression or treatment of PC. However, further in-depth investigation is required to fully understand how miRNAs are involved in the development and progression of PC, and finding which agents including natural compounds and how they could regulate the expression of distinct miRNAs. The answers will help us to design novel treatment strategies for the cure of PC patients.
